# Comment on “The impact of drug eluting stents on the co-release of interleukin-6 in patients with stable angina”

**DOI:** 10.21542/gcsp.2025.18

**Published:** 2025-05-15

**Authors:** Hinpetch Daungsupawong, Viroj Wiwanitkit

**Affiliations:** 1 Private Academic Consultant, Phonhong, Lao People’s Democratic Republic; 2 Department of Research Analytics, Saveetha Dental College and Hospitals, Saveetha Institute of Medical and Technical Sciences, Saveetha University, Chennai, India

Dear Editor,

We would like to comment on “The impact of drug eluting stents on the co-release of interleukin-6 in patients with stable angina^1^.” The purpose of this study was to evaluate the immediate effects of drug-eluting stents (DES) on the inflammatory response in stable angina patients by measuring interleukin-6 (IL-6), a marker of systemic inflammation. The findings revealed an increase in IL-6 levels both immediately and 24 h after stent implantation. However, these findings raise several significant questions about the processes and implications of stent use in individuals with stable angina.

The first point that may spark debate is whether there is a link between elevated IL-6 and the likelihood of long-term problems from drug-eluting stent use, such as recurring stenosis or possibly deadly thrombosis. Although the results show an increase in IL-6, it is unknown how this alteration affects disease progression or long-term repercussions. More research is needed to determine the long-term influence of these cytokine alterations on key outcomes.

The second concern is why statins may reduce IL-6 levels in some patients at first but then decrease following stent placement? Statins were observed to lower IL-6 levels initially, but not considerably after stent installation. This could represent the intricacies of the immunological or inflammatory processes that occur following stent implantation, and more research in this area could help us better understand the function of statins and their methods of action.

Another relevant question is if the amount of inflammation after stent insertion differs by gender. In this study, men had a significantly higher IL-6 level than women following stent insertion. This could be due to immune-related changes or hormonal impacts. This opens the door to future in-depth research into sex variations in inflammatory processes and the possible impact of stent treatment.

Finally, the small sample size is a significant constraint that may preclude the findings from being applied to a larger population. To generate more trustworthy and therapeutically useful results, larger sample sizes should be used in future investigations.

Further research into the impacts of stent release and long-term inflammatory responses will help us better understand the mechanisms that contribute to the development of treatment-related problems and how to prevent them.
